# The Voluntary Hospitals and the Insurance Bill

**Published:** 1911-07-29

**Authors:** 


### The Hospital

A Journal of

The Medical Sciences and Hospital Administration.

New Series. No. 235, Vol. IX. [No. 1307, Vol. L.] SATURDAY, JULY 29, 1911

### The Voluntary Hospitals and the Insurance Bill.

Our present issue contains the report of a deputation to Mr. Lloyd George representing the British Hospitals Association, the Central Hospital Council for London, and the London Hospital Saturday Fund. We further publish a useful memorandum, prepared by the voluntary hospitals in Scotland, as adopted at a meeting held in Glasgow on July 21. The latter memorandum vigorously endorses the recommendations we made last week. Mr. Lloyd George's reply to the deputation was on the whole sympathetic. It appears that the omission of the voluntary hospitals from the Bill was deliberate because, as the Chancellor puts it, he had not legislated for these hospitals since he believed in the voluntary system and wanted it to continue. Welcoming this indication of his opinion on the question of voluntary hospitals, we very earnestly hope the Chancellor will give practical effect to it by amending Clause 15 of the Bill as drafted to the extent we suggested last week. The amended clause would then run as follows: "15 (1) For the purposes of administering institutional benefit, local Health Committees shall make arrangements to the satisfaction of the Insurance Commissioners with persons or local authorities having the management of hospitals, sanatoria, or other similar institutions approved by the Local Government Board for the treatment therein of insured persons entitled to institutional benefit under this part of the Act." Such an amendment would give the local Health Committees power to make arrangements with the managers of voluntary hospitals for the treatment of insured persons upon a plan which would preserve the voluntary principle of hospital support and protect these institutions from much of the financial injury which must otherwise be caused by the Bill. Then, if Mr. Lloyd George will further provide that the voluntary hospitals for each locality shall be represented upon the local Health Committees, he will in a very simple way have set up machinery which, with the co-operation of the voluntary hospitals, can be made effective to remove the present abuses attaching to them and secure the best treatment for all classes of people affected by the Insurance Bill in a way which must conserve all the interests concerned.

Various suggestions were made by the deputation. Mr. Lloyd George agreed that some contribution from the insurance fund should be made available to defray the cost of the work which the hospitals might be called upon to render towards insured persons. That is a practical detail which we can well leave with the Chancellor, providing always that care is taken to protect the voluntary hospitals by taking this money out of the insurance fund as a whole without any regard to the contributions of individual patients. Such payments must of course come from the insurance fund, but they will be paid at the instigation of the local Health Committees, and will be subject to an arrangement or contract made between these bodies and the voluntary hospital authorities. Care must be taken, however, that all such contracts shall be based upon a *pro rata* payment for actual work done for the State at the request of the local Health Committees. In this way, by reorganising the distribution of beds, it will be possible for a voluntary hospital to accept such payments, and to maintain its position as a voluntary hospital pure and simple.

There can be no doubt as to the financial damage which the Bill, as drafted, if enacted, must cause to the voluntary hospitals. Mr. Lloyd George stated that in his view members of Friendly Societies would be relieved of their present payment to such bodies to the extent of from 6d. to 1s. a month. This saving to them, he seemed to consider, would be likely to be given by the members of Friendly Societies to aid the voluntary hospitals, but our experience would lead us to an opposite conclusion. Members of Friendly Societies, as such, have not been in the habit of contributing materially to hospitals in the past, and we do not suppose that, when they are compelled to contribute regularly a certain sum to a State insurance scheme, they will be likely to change their present attitude by becoming ardent and liberal supporters of the voluntary hospitals. Of course, everybody who thinks must recognise the voluntary hospitals to be an essential part of the machinery of civilisation. People are willing enough to admit this fact, but when it comes to voluntary gifts experience shows that a very small percentage of the whole population, in fact, contribute anything, even casually, to the support of our voluntary hospitals. The burden of support has been cheerfully borne by a small minority of citizens who regard it as a high privilege in the days of health to do their utmost to provide for their poorer neighbours in the hour of sickness or accident. We note and shall remember Mr. Lloyd George's declaration that no country or Government can possibly allow a hospital to be closed because it is short of cash. The history of the world affords instances to the contrary, but we look to Mr. Lloyd George with confidence to render such a thing impossible so far as the effects of his insurance legislation is concerned, by amending Clause 15 to the extent we have suggested. That will be giving practical effect to his opinion and wishes, and at the same time provide reasonable safeguards for the voluntary hospitals, whilst it will do a national service by giving an impetus to the reorganisation of our present methods of hospital relief and their consequent improvement in many directions.

We believe that the amendment of Clause 15 may readily be made to pave the way to a thorough reform of the abuses attaching to our existing hospital system, whilst it should enable every penny of the money available for hospital benefit to be economically expended to the best advantage of everybody concerned. It should lead to an active co-operation among all agencies for hospital care and sick benefit upon a plan which has had material influence for good in several great American cities, and has not been without its value even in London. Mr. Lloyd George is now earnestly considering the changes he will introduce into his Bill, so far as it affects hospitals, and he asked the deputation to arrange that the managers of the great voluntary hospitals should talk over the questions here raised with the medical profession. We hope that the voluntary hospital managers in great cities, and especially those which have medical schools associated with them, will call together the members of their honorary staff and confer with them in compliance with Mr. Lloyd George's request.

